# The Intestinal Barrier in Irritable Bowel Syndrome: Subtype-Specific Effects of the Systemic Compartment in an *In Vitro* Model

**DOI:** 10.1371/journal.pone.0123498

**Published:** 2015-05-15

**Authors:** Samefko Ludidi, Daisy Jonkers, Elhaseen Elamin, Harm-Jan Pieters, Esther Schaepkens, Paul Bours, Joanna Kruimel, José Conchillo, Ad Masclee

**Affiliations:** Division of Gastroenterology-Hepatology, Department of Internal Medicine, NUTRIM School for Nutrition, Toxicology and Metabolism-Maastricht University Medical Center+, Maastricht, The Netherlands; Hungarian Academy of Sciences, HUNGARY

## Abstract

**Background:**

Irritable bowel syndrome (IBS) is a disorder with multifactorial pathophysiology. Intestinal barrier may be altered, especially in diarrhea-predominant IBS (IBS-D). Several mediators may contribute to increased intestinal permeability in IBS.

**Aim:**

We aimed to assess effects of tryptase and LPS on *in vitro* permeability using a 3-dimensional cell model after basolateral cell exposure. Furthermore, we assessed the extent to which these mediators in IBS plasma play a role in intestinal barrier function.

**Materials and Methods:**

Caco-2 cells were grown in extracellular matrix to develop into polarized spheroids and were exposed to tryptase (10 - 50 mU), LPS (1 - 50 ng/mL) and two-fold diluted plasma samples of 7 patients with IBS-D, 7 with constipation-predominant IBS (IBS-C) and 7 healthy controls (HC). Barrier function was assessed by the flux of FITC-dextran (FD4) using live cell imaging. Furthermore, plasma tryptase and LPS were determined.

**Results:**

Tryptase (20 and 50 mU) and LPS (6.25 – 50 ng/mL) significantly increased Caco-2 permeability versus control (all *P*< 0.05). Plasma of IBS-D only showed significantly elevated median tryptase concentrations (7.1 [3.9 – 11.0] vs. 4.2 [2.2 – 7.0] vs. 4.2 [2.5 – 5.9] μg/mL; *P*<0.05) and LPS concentrations (3.65 [3.00 – 6.10] vs. 3.10 [2.60-3.80] vs. 2.65 [2.40 – 3.40] EU/ml; *P*< 0.05) vs. IBS-C and HC. Also, plasma of IBS-D increased Caco-2 permeability versus HC (0.14450 ± 0.00472 vs. 0.00021 ± 0.00003; *P* < 0.001), which was attenuated by selective inhibition of tryptase and LPS (*P*< 0.05).

**Conclusion:**

Basolateral exposure of spheroids to plasma of IBS-D patients resulted in a significantly increased FD4 permeation, which was partially abolished by selective inhibition of tryptase and LPS. These findings point to a role of systemic tryptase and LPS in the epithelial barrier alterations observed in patients with IBS-D.

## Introduction

Irritable bowel syndrome (IBS) is a functional gastrointestinal (GI) disorder, with an estimated prevalence of 10–15% in Western countries [1]. It is characterized by chronic abdominal pain or discomfort, and altered bowel habits in the absence of an organic cause [2].

IBS is a heterogeneous disorder of multifactorial origin with various disturbances along the gut-brain axis [3]. Recently, attention has been paid to low-grade chronic inflammation, changes in the composition of the intestinal microbiome [4–7], motility and visceral perception [3, 8]. Furthermore, alterations in the intestinal barrier have been reported in patients with IBS [9, 10] and were found to be associated with GI symptoms, such as diarrhea and abdominal pain [5]. It has been hypothesized that barrier dysfunction is an early event in IBS and may contribute to low-grade intestinal inflammation and increased visceral perception [11]. Studies that evaluated intestinal permeability in IBS subtypes reported that especially patients with diarrhea-predominant IBS (IBS-D) [12, 13] and post-infectious (PI) IBS frequently show altered intestinal barrier function [14].

Altered epithelial integrity may result from both intestinal and systemic factors. Inflammatory mediators, *e*.*g*. mast cell products, may contribute to impaired intestinal barrier function in IBS [15]. Tryptase (TRYP) is a serine protease, released by mast cells upon degranulation. An increased release of TRYP has been reported in patients with IBS and is more prominent in patients with IBS-D compared to patients with constipation predominant IBS (IBS-C) [16–18]. Luminal exposure to TRYP affects intestinal paracellular permeability both *in vitro* [15] and *ex vivo* [19]. Recent data indicate that faecal supernatants and mucosal homogenates of IBS-D patients increase permeability in *ex vivo* models of intestinal tissue in Ussing chambers [17, 19].

Alterations in barrier function as observed in IBS, may give rise to permeation of bacteria and their products. Lipopolysaccharide (LPS), the major component of the cell wall of Gram-negative bacteria, is also able to affect barrier function. Under physiological conditions LPS will not pass the intact epithelium, but a reduced epithelial integrity may allow LPS to pass the GI barrier [20, 21].

Although both TRYP and LPS can be found in the systemic compartment, little is known about basolateral exposure relative to luminal exposure of these modulators in relation to barrier function. Furthermore, the question arises whether these systemic factors may be involved in the altered intestinal permeability in patients with IBS and secondly, whether differences exist between IBS subtypes. We hypothesize that TRYP and LPS in the systemic compartment contribute to the altered intestinal permeability as observed in IBS patients with diarrhea

Recently, we have shown that a three-dimensional (3D) cell culture model of Caco-2 spheroids represents a highly suitable physiological model to investigate basolateral exposure of substances on paracellular permeability [22, 23]. Aim of the present explorative study was 1) to evaluate the effect of basolateral exposure of TRYP and LPS on intestinal permeability in the 3D intestinal cell culture model 2) to assess TRYP and LPS plasma levels in patients with IBS-D and IBS-C versus healthy controls (HC) 3) to investigate the effect of plasma of IBS-D and IBS-C patients on intestinal permeability *in vitro* and 4) whether such an effect is TRYP and/or LPS related.

## Materials and Methods

### Cell line, culture conditions and three-dimensional (3D) cell culture

Colonic adenocarcinoma cell line (Caco-2) from the American Type Culture Collection (ATCC, Rockville, USA) were maintained in Dulbecco’s Modified Eagle Medium (DMEM; Lonza Benelux BV, Breda, the Netherlands) and cultured in 3D as described previously (22). Briefly, Caco-2 cells (4x10^4^ cells/ dish; passage 26–36) were embedded in 40% growth factor-reduced Matrigel (8 mg/ml; BD Biosciences, San José, CA—USA) and solidified at 37°C for 30 min in glass bottom culture dishes. Thereafter, medium was added and spheroids were allowed to form over 5–7 days at 37°C [22].

### Exposure to TRYP and LPS and determination of intestinal epithelial barrier function

3D spheroids were exposed basolaterally to TRYP concentrations of 10, 20 and 50 mU (Sigma-Aldrich, Chemie B.V., Zwijndrecht, The Netherlands), LPS concentrations of 1, 6,25, 12.5, 25 and 50 ng/mL (Sigma-Aldrich) and combinations of TRYP and LPS (10 mU TRYP + 6.25 ng/mL LPS and 20 mU TRYP + 12.5 ng LPS) in the presence of 10% (v/v) fluorescein isothiocyanate-labelled dextran of 4 kDa (FD4; Sigma-Aldrich) at 37°C for 24 hours. Two mM ethylene glycol tetra acetic acid (EGTA) was used to induce maximum TJs disruption and to serve as positive control. Growth medium only was used as negative control. Permeability of the spheroids was determined by the flux of FD4 from the basal to the luminal compartment (*i*.*e*., L/BL ratio) using confocal microscopy as described previously [23]. Confocal images were taken with Leica TCS SPE confocal laser scanning microscope (Leica Microsystems GmbH, Mannheim, Germany) and processed using TCS SPE browser and Image J software [24].

Thereafter, spheroids were exposed to TRYP (10, 20 and 50 mU) or LPS (1, 6.25 and 12.5 ng/mL) in the presence of either 100 μM TRYP inhibitor nafamostat mesylate (NM) [19] or LPS inhibitor polymyxin B sulphate salt (PMB) at 10, 62.5 or 125 ng/mL [25], respectively. Spheroids were also exposed to TRYP and LPS in combination with NM and PMB, followed by assessment of basal to luminal permeation of FD4.

### Assessment of cell viability

Cell viability was determined by measuring release of lactate dehydrogenase (LDH), an indicator of plasma membrane integrity, using the LDH assay (CytoTox ONE Homogeneous Membrane Integrity Assay; Promega, the Netherlands). Briefly, Caco-2 cells were cultured in 3D in 96-well plates and incubated with 200 μL of solutions containing LPS (all concentrations), TRYP (all concentrations) and the combination of TRYP and LPS (i.e.10 mU TRYP in combination with 1 ng/ml LPS and 20 mU TRYP in combination with 6.25 ng/ml LPS) with and without their respective inhibitors. Plates were incubated at 37°C for 24 hours and equilibrated at room temperature (RT) for 20 min. Then of the substrate mix was added and incubated at RT, protected from light for another 30 minutes, followed by addition of 20 μL stop solution. Maximum LDH release was induced by using 200 μL of 1% (v/v) Triton with 99% (v/v) PBS (Sigma-Aldrich, Chemie B.V., Zwijndrecht—The Netherlands). The fluorescence was measured at an excitation and an emission wavelength of 560 nm and 590 nm, respectively. The percentage of LDH activity was calculated as percentage of maximum LDH release and compared with negative control.

### Study population and sample collection

Patients with clearly defined IBS-D (N = 7) and IBS-C (N = 7) subtypes, according to the Rome III criteria, and 7 HC were included in the present study. From each subject, blood samples were collected in pre-cooled Sodium-Heparin tubes and immediately placed on ice. Within 2 hours, plasma samples were centrifuged (4000 rpm; 4°C) after which the supernatants were collected and stored at -80°C until further experiments.

The study was part of a large prospective cohort study in IBS patients. All subjects had signed an informed consent prior to participation. The study protocol has been approved by the Maastricht University Medical Centre+ Committee of Ethics and was executed according to the Declaration of Helsinki (59^th^ general assembly of the WMA, Seoul, South Korea, Oct. 2008). The study has been registered in the US National Library of Medicine (*NCT00775060*).

### Quantification of TRYP and LPS concentrations in plasma

Total TRYP levels in plasma were measured using the ImmunoCAP (Phadia AD, Upsala, Sweden). The enzyme-based Limulus amoebocyte lysate chromomeric endpoint assay (Hycult Biotech, Uden, The Netherlands) was used to measure plasma LPS concentrations. Both were performed according to manufacturer’s instructions.

### Exposure of human plasma on intestinal epithelial barrier function *in vitro*

To assess barrier function, 3D spheroids were incubated basolaterally with the plasma samples of the IBS-D, IBS-C and HC. Medium only and 2 mM ethylene glycol tetra-acetic acid (EGTA) were used as negative and positive controls, respectively. Spheroids were incubated with 2 mL containing 37.5% (v/v) plasma, 52.5% (v/v) medium and 10% (v/v) FD4. The basal to luminal FD4 permeation was assessed by confocal microscopy as described above.

Subsequently, effects of NM (100 μM) and PMB (62.5 ng/mL) were assessed on *in vitro* permeability after co-incubation with plasma of the IBS-D patients.

### Data analyses

All experiments were performed in triplicate and results were reported as mean ± SEM of at least 5 spheroids per experiment and 7 z stack measurements per spheroid. *In vitro* experiments were performed with plasma samples of 7 subjects in each subgroup (*i*.*e*. HC vs. IBS-D vs. IBS-C). Permeability data were analysed parametrically using a one-way analysis of variance (ANOVA) and Tukey’s post-hoc test was performed to determine significant differences between different treatment conditions (GraphPad Software Incorporated, CA, USA—version 6.0; Macintosh).

Serine protease activity and LPS concentrations in plasma were compared between groups using a Kruskal-Wallis test. A *P*-value under 0.05 was considered statistically significant. A Bonferroni correction for multiple testing was applied to all data.

## Results

### Subjects

IBS patients were selected based on IBS-subtype (IBS-D (N = 7) or IBS-C (N = 7)) and compared with HC (N = 7). The IBS groups did not differ significantly with regards to disease duration and age, but female sex was significantly more prevalent in IBS-C versus IBS-D (85.7% vs. 28.6%, respectively; *P* < 0.05). No differences were found in age and gender comparing HC with the IBS subgroups.

### FD4 permeation after basolateral exposure to TRYP and LPS

**Fig 1:**
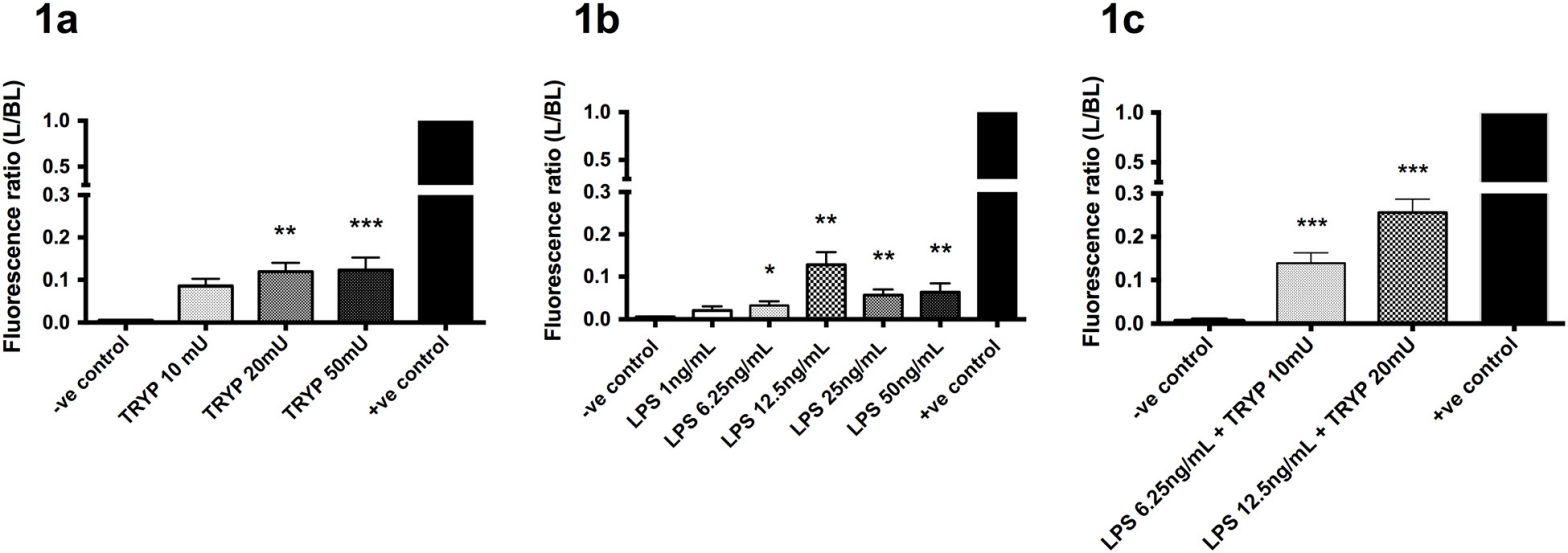
Permeation of FD4 (mean ± SEM) after exposure to different concentrations of TRYP (1a); LPS (1b) and combination of TRYP and LPS (1c). *P < 0.05; **P < 0.01; ***P < 0.001 compared to negative control (medium only). Analyses were based on at least 5 spheroids per experiment. Experiments were carried out three independent times.

TRYP at 20 mU and 50 mU induced a significant increase of the basal to luminal FD4 flux (Fig 1a, *P* < 0.01 and *P* < 0.001, respectively). A non-significant increase was found for 10 mU TRYP (*P* = 0.076).

Exposure to LPS also increased FD4 permeation at concentrations of 6.25 ng/mL (*P* < 0.05), 12.5 ng/mL, 25 ng/mL and 50 ng/mL (all *P* < 0.01), but no effect was found for 1 ng/mL (Fig 1b). Furthermore, when compared to negative control, the combination of 10 mU TRYP with 6.25 ng/mL LPS as well as 20 mU TRYP with 12.5 ng/mL LPS significantly increased the L/BL FD4 ratio versus negative control (Fig 1c; both *P* < 0.001), which was also significantly higher compared to each of the stressors separately (*P* < 0.01), pointing to an additive effect of TRYP and LPS combined.

### TRYP and LPS levels in plasma

**Fig 2:**
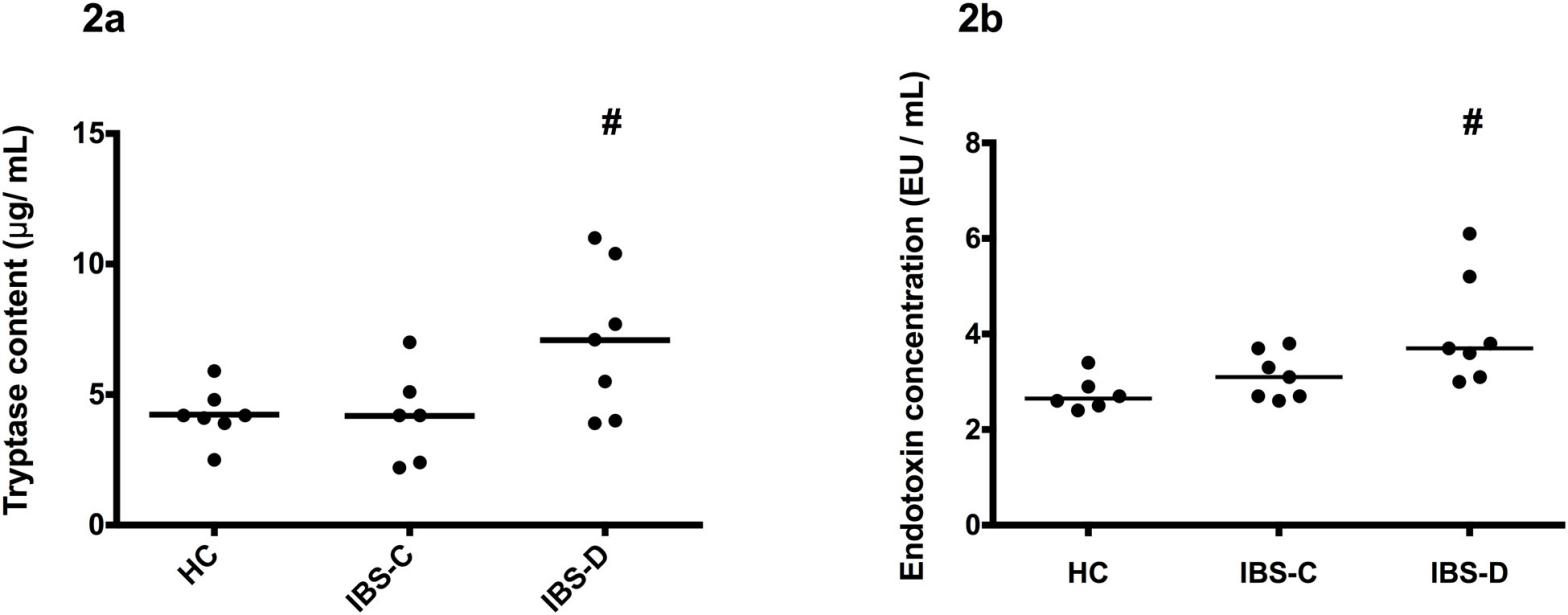
Median plasma TRYP (2a) and LPS levels (2b) in healthy controls (HC, n = 7 and n = 6, respectively), constipation-predominant (IBS-C, n = 7) and diarrhea-predominant IBS patients (IBS-D, n = 7); 2a #P < 0.05 compared to HC and IBS-C; 2b #P < 0.05 compared to HC only. Experiments were carried out three independent times.

Median TRYP levels were significantly higher in plasma of IBS-D versus HC and IBS-C (Fig 2a; 7.1 [3.9–11.0] vs. 4.2 [2.5–5.9] vs. 4.2 [2.2–7.0] ng/mL, respectively; *P* < 0.05). Furthermore, LPS levels were significantly increased in plasma of IBS-D patients compared to HC (3.65 [3.00–6.10] vs. 2.65 [2.40–3.40] EU/ml, respectively; *P* < 0.05), but not compared to IBS-C (3.10 [2.60–3.80] EU/ml; *N*.*S*.). No significant differences were found between IBS-C and HC (Fig 2b).

### Inhibition of TRYP and LPS on FD4 permeation

Compared to 20 mU TRYP alone, adding the TRYP inhibitor NM (100 μM) significantly attenuated the TRYP-induced increase of the L/BL FD4 ratio (0.0541 ± 0.013 vs. 0.1139 ± 0.021; *P* < 0.05). A similar effect was observed after exposure to 50 mU TRYP with 100 μM NM versus 50 mU TRYP only (0.0513 ± 0.012 vs. 0.1231 ± 0.029; *P* < 0.01). No significant effect was observed when adding 100 μM NM to 10 mU TRYP.

Concentrations up to 25 ng/mL LPS were tested for co-incubation with PMB. Only simultaneous exposure to 12.5 ng/mL LPS with 125 ng/mL LPS inhibitor PMB, resulted in a significant decrease versus 12.5 ng/mL LPS alone (0.0534 ± 0.010 vs. 0.128 ± 0.030; *P* < 0.05). No significant effects were observed for the other concentrations of LPS after co-incubation with their respective concentrations of PMB.

A slight but significant increase in FD4 permeation was also found following separate exposure to NM and PMB, compared to the negative control (0.0737 ± 0.025 and 0.0358 ± 0.023, respectively, versus 0.00424 ± 0.0011; both *P* < 0.0001).

### TRYP, LPS and cell viability

No significant increase in LDH release as indicator of plasma membrane integrity, hence cell viability, was detected after incubation with TRYP (10 and 20 mU), LPS (1 to 25 ng/ml, nor for, the combination of TRYP and LPS with or without their respective inhibitors NM and PMB (all *P* > 0.05).

### FD4 permeation in 3D spheroids after exposure to plasma

**Fig 3:**
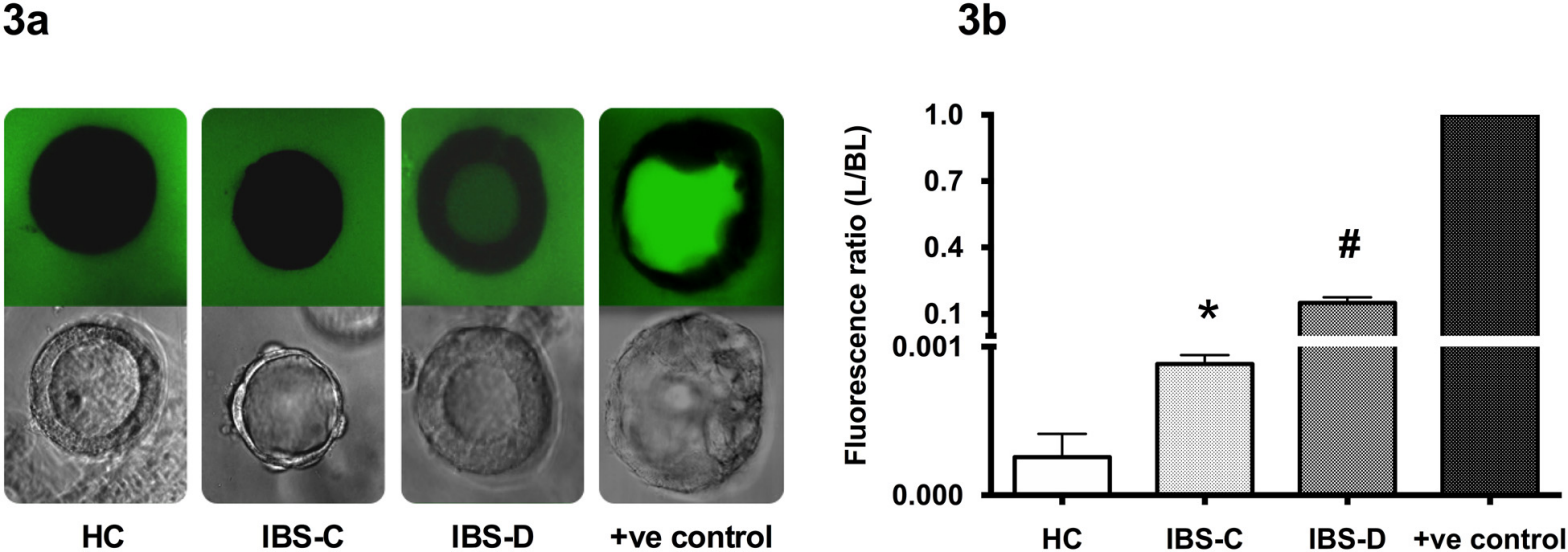
Permeation of FD4 after exposure to plasma of healthy controls (HC, n = 7), constipation-predominant IBS (IBS-C, n = 7), diarrhea-predominant IBS (IBS-D, n = 7) and positive control (+ve control i.e. EGTA) using confocal microscopy (3a) and expressed as mean ± SEM L/BL fluorescence ratio of triplicate experiments (3b) *P < 0.0001 vs. HC #P < 0.0001 vs. HC and IBS-C. Experiments were carried out three independent times.

In Fig 3, the FD4 flux after basolateral exposure to plasma has been shown. Spheroids exposed to plasmas of IBS-D as well as IBS-C showed significant increased L/BL FD4 ratios in comparison to HC. The magnitude of the effect was significantly larger in IBS-D, showing increased L/BL FD4 ratios in comparison with plasma of IBS-C (*P* < 0.0001).

**Fig 4:**
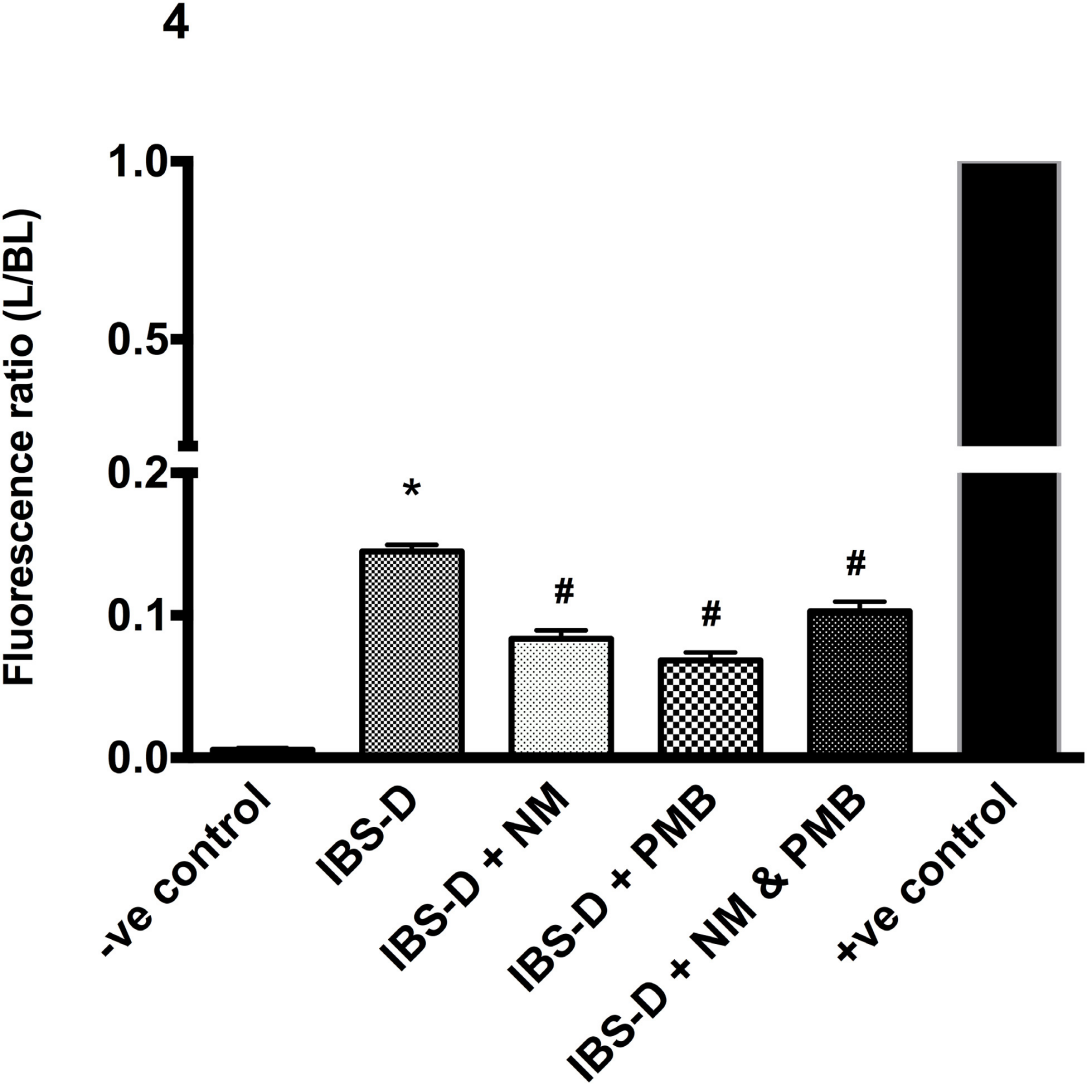
Permeation of FD4 (mean ± SEM), expressed as L/BL fluorescence ratio after exposure to plasma of patients with diarrhea-predominant IBS (IBS-D); plasma of IBS-D with 100uM nafamostat mesylate (NM); plasma of IBS-D with 62.25 ng/mL polymyxin B (PMB) and a combination of plasma, NM and PMB (*P < 0.0001 vs. HC; #P < 0.0001 vs. IBS-D). Experiments were carried out three independent times.

Furthermore, the increased FD4 permeation found after exposure to plasma of IBS-D patients, was significantly reduced after co-incubation with NM (100 μM), PMB (62.5 ng/mL) or the combination of both (Fig 4: all *P* < 0.0001).

## Discussion

Using the 3D intestinal cell culture model, we found that basolateral exposure to both tryptase and LPS induced a significant increase in FD4 permeation. Plasma LPS and tryptase levels were increased IBS-D, but not in the IBS-C patients. A significant increase in FD4 permeation was observed when 3D spheroids were exposed to plasma of IBS-D compared to IBS-C patients and controls. These effects were partially abolished by adding tryptase- and LPS-inhibitors.

Recent data suggest that local inflammation is associated with alterations in intestinal barrier function [13, 26, 27]. Low-grade intestinal inflammation has repeatedly been demonstrated in IBS [28] and findings point to a subtype- specific increase of tryptase in mucosal and faecal samples [16, 18, 29] and to a key role for mast cells and tryptase in the altered barrier function of IBS-D patients [17, 19]. Tryptase can induce signal transduction via protease-activated receptors (PAR), which are abundantly expressed in intestinal epithelium [30, 31]. Their activation may result in increased paracellular permeability and visceral perception [17].

Whereas most studies focused on apical or luminal intestinal cell surface exposure, little is known on basolateral exposure. Up to now Lee *et al*. were the only group to report that tryptase increased permeability when added to the basolateral surface of intestinal biopsies of healthy subjects [19].

We employed a three-dimensional Caco-2 cell culture model to promote a physiologically relevant microenvironment mimicking the specificity of tissues *in vivo*, with fully polarized cells including formation of tight and adherens junctions and a central lumen. Unlike the conventional two-dimensional, cultures in extracellular matrix (ECM) proteins form 3D spheroids and re-establish cell–ECM, cell–cell interactions and signaling pathways involved in cell differentiation, proliferation and barrier function [32]. Currently 3D cultures are widely used in cell biology studies, including cell adhesion, cell migration, tumour biology, and epithelial morphogenesis [33]. The model is found to be highly suitable to study epithelial barrier function after basolateral exposure [22, 23]. The overall results were in line with those obtained by the 2D model [34, 35].

Using the 3D sphehoids, we were able to show that tryptase and LPS, even at low concentrations (*i*.*e* 20mU tryptase and 6.25–12.5 ng/mL LPS) can result in increased FD4 permeation, indicating enhanced paracellular permeability. In addition, we found a significant increase in FD4 permeation after exposure to plasma of both IBD-D and IBS-C patients compared to HC, with a significantly higher effect for IBS-D versus IBS-C patients. Using this approach, we could confirm the *in vitro* findings by Wilcz-Villega *et al*. [36] and by Lee *et al*. using biopsies of healthy subjects mounted in Ussing chambers [19]. Moreover, we expanded on their findings by demonstrating for the first time significantly elevated tryptase and LPS concentrations in plasma of IBS-D patients comparison to HC. Furthermore, plasma tryptase was also significantly increased in IBS-D compared to IBS-C patients. Our data indicate that plasma tryptase may contribute to the changes in barrier function observed after exposure to plasma of IBS-D patients, since selective inhibition of plasma tryptase significantly reduced intensity of FD4 permeation. Increased numbers and/or activity of mast cells as observed in patients with IBS-D, may reflect tryptase release and spill-over from the intestinal compartment into the systemic circulation, contributing to the observed differences between IBS-D, HC and IBS-C [37].

The second mediator we investigated was basolateral exposure to LPS, which significantly and dose-dependently increased FD4 permeation (from the basolateral side into the lumen). This observation is in line with *in vitro* studies showing increased permeability of Caco-2 monolayers after basolateral exposure to high (100–500 μg/mL) [38] but also low (0.3–20 ng/mL) [39] concentrations of LPS. Remarkably, 12.5 ng/mL LPS caused a significant peak in FD4 permeation in all experiments performed in triplicate. It is not known what causes this dose-specific difference, since the LDH essay could not reveal decreased cell viability at concentrations of of 12.5 ng/mL LPS. It has to be noted however that, in our study, cell viability was assessed using an LDH assay. In this assay, cellular toxicity is determined based on disruption of plasma membrane integrity and subsequently, LDH release. Although LDH leakage was not significantly affected at any of the LPS concentrations used, this method underestimates cytotoxicity induced by toxic agents that could influence intracellular activities. In future studies, additional tests based on apoptosis markers such caspase 3/7 activation and cytokeratin 18 cleavage are required to complement the LDH assay.

Elevated LPS concentrations (*i*.*e*. about 1.5-fold increase) have previously been found in patients with inflammatory bowel disease when compared to healthy subjects, and were also found to be related to disease activity [40]. In the present study, circulating LPS concentrations were also found to be significantly increased 1.5 fold in IBS-D patients compared to HC. Although LPS can permeate to the systemic compartment as a consequence of a disturbed barrier function, it is important to note that LPS present in the circulation can also affect barrier function and contribute to sustain an increased permeability from the basolateral side. In line with our observations and hypothesis, O’Dwyer *et al*. demonstrated that a single dose of 4 ng/mL LPS infused intravenously was able to increase intestinal permeability in healthy subjects [41].

Although concentrations for tryptase and LPS were increased in IBS-D, they were still within the physiological range. However, the combination of tryptase and LPS was found to induce a more pronounced effect on *in vitro* permeability than each mediator separately, pointing to an additive effect. This may (in part) explain the significant increase in BL/L FD4 flux after adding plasma of IBS-D, despite the relative low concentrations of tryptase and LPS. When selectively inhibiting tryptase and LPS after plasma exposure, permeability showed a significant, but incomplete decrease. This might be the result of a relative overdose of nafamostat mesylate, as that the tryptase inhibitor alone showed an effect on the BL/L FD4 flux. Furthermore, we should acknowledge the influence of other yet not identified mediators.

Plasma of IBS-D patients resulted in markedly and significantly increased FD4 permeation compared to HCs and IBS-C patients, which, at least partially, was related to elevated tryptase and LPS levels. However, the plasma of IBS-C patients also showed a significant but much smaller increase in FD4 permeation when added to the 3D spheroids. LPS and tryptase levels were not found to be increased in these patients, suggesting that other factors, such as cysteine protease activity [16] may have induced the observed effect. Considering the rather small increment in permeability caused by plasma of IBS-C patients in our study, we think it is unlikely that substantial cysteine protease activity could have contributed to the permeability alterations observed in neither IBS-C nor IBS-D patients. These data encourages the hypothesis that the IBS subtypes with predominant diarrhea and constipation are different entities, not only in clinical presentation, but also regarding the underlying pathophysiological mechanisms.

One of the major weaknesses of our study is that we did not take into account the potential effect of medication that may affect permeability [42], such as proton pump inhibitors (PPI) and non-steroidal anti-inflammatory drugs (NSAIDs) medications [43]. Although all subjects participating in our study were asked to refrain from any medication 24 hours before blood collection, chronic or long-term medication intake may have had an effect on intestinal permeability in our patients as well.

The strength of the present study is that we aimed to translate the *in vitro* permeability findings after basolateral exposure of tryptase and LPS, to the *in vivo* situation by incubating the 3D spheroids with plasma samples of patients and controls and selectively blocking potential mediators to confirm their contributing role. Although it has to be acknowledged than only a limited number of subjects were included per group, the experiments were performed in triplicate and all individual plasma samples of IBS-D patients induced an increased intestinal permeability. However, one must realize that effects observed in Caco-2 cells cannot be one to one extrapolated to the *in vivo* situation. Nonetheless, since cell-to-cell interaction is crucial in epithelial cell biology [44], the 3D model provides a more realistic microenvironment to simulate *in vivo* responses to stressors compared to 2-dimensional monolayers [22, 45]. Furthermore, in the present study, all analyses were done at a functional level by measuring FD4 permeation as indicator of paracellular permeability. No structural analyses have been performed investigating for example the localization and expression of tight junction (TJ) proteins. As tryptase and LPS can induce TJ re-localization [17, 39], analyses of TJs and signaling cascades may provide additional insight in the mechanisms involved.

In summary, we demonstrated that basolateral exposure of a 3D cell model to tryptase and LPS significantly altered FD4 permeation. In plasma of patients with IBS-D, circulating tryptase and LPS were significantly increased andexposure of the spheroids to plasma of these patients resulted in increased FD4 permeation. The magnitude of the increased permeability was significantly more pronounced in IBS-D than IBS-C patients and selective inhibition of tryptase and LPS partially abolished the effect. These data point to a role of systemic tryptase and LPS in epithelial barrier dysfunction in patients with IBS-D.
